# Increased Expression of PcG Protein YY1 Negatively Regulates B Cell Development while Allowing Accumulation of Myeloid Cells and LT-HSC Cells

**DOI:** 10.1371/journal.pone.0030656

**Published:** 2012-01-23

**Authors:** Xuan Pan, Morgan Jones, Jie Jiang, Kristina Zaprazna, Duonan Yu, Warren Pear, Ivan Maillard, Michael L. Atchison

**Affiliations:** 1 Department of Animal Biology, School of Veterinary, University of Pennsylvania, Medicine, Philadelphia, Pennsylvania, United States of America; 2 Center for Stem Cell Biology, Life Sciences Institute, Departments of Medicine & Cell and Developmental Biology, University of Michigan, Ann Arbor, Michigan, United States of America; 3 Division of Hematology, The Children's Hospital of Philadelphia, Philadelphia, Pennsylvania, United States of America; 4 Department of Pathology and Laboratory Medicine, School of Medicine, University of Pennsylvania, Philadelphia, Pennsylvania, United States of America; Centro Cardiologico Monzino, Italy

## Abstract

Ying Yang 1 (YY1) is a multifunctional Polycomb Group (PcG) transcription factor that binds to multiple enhancer binding sites in the immunoglobulin (Ig) loci and plays vital roles in early B cell development. PcG proteins have important functions in hematopoietic stem cell renewal and YY1 is the only mammalian PcG protein with DNA binding specificity. Conditional knock-out of YY1 in the mouse B cell lineage results in arrest at the pro-B cell stage, and dosage effects have been observed at various YY1 expression levels. To investigate the impact of elevated YY1 expression on hematopoetic development, we utilized a mouse in vivo bone marrow reconstitution system. We found that mouse bone marrow cells expressing elevated levels of YY1 exhibited a selective disadvantage as they progressed from hematopoietic stem/progenitor cells to pro-B, pre-B, immature B and re-circulating B cell stages, but no disadvantage of YY1 over-expression was observed in myeloid lineage cells. Furthermore, mouse bone marrow cells expressing elevated levels of YY1 displayed enrichment for cells with surface markers characteristic of long-term hematopoietic stem cells (HSC). YY1 expression induced apoptosis in mouse B cell lines in vitro, and resulted in down-regulated expression of anti-apoptotic genes Bcl-xl and NFκB2, while no impact was observed in a mouse myeloid line. B cell apoptosis and LT-HSC enrichment induced by YY1 suggest that novel strategies to induce YY1 expression could have beneficial effects in the treatment of B lineage malignancies while preserving normal HSCs.

## Introduction

Yin Yang 1 (YY1) is a ubiquitous and multifunctional zinc-finger transcription factor that mediates multiple diverse functions. YY1 can act as a transcriptional activator, repressor or initiator protein depending upon DNA binding site context or cell type [1], [2], [3]. Homozygous disruption of the *yy1* gene in mice results in peri-implantation lethality. Heterozygous knock-out mice show growth retardation and some neurological defects [4]. Reduction of YY1 levels impairs embryonic growth and viability in a dose-dependent manner [5]. There is a tight correlation between YY1 dosage and cell proliferation, with deletion of the *yy1* gene resulting in cytokinesis failure and cell cycle arrest [5]. YY1 is also implicated in lineage differentiation and cell growth control [6], [7], [8], as well as in oncogenesis and other diseases such as dystrophic muscle disease [6], [9].

YY1 can bind the retinoblastoma (Rb) protein to accelerate cell cycle progression to the S phase [8], [10], can activate c-myc P1 promoter activity in Burkitt's lymphoma [11], and can enhance murine double minute 2 (mdm2)-mediated p53 inactivation thus potentiating cellular proliferation and tumorigenesis [12]. On the contrary, in human basal cell carcinoma YY1 shows repressive activity at the GST locus and may prevent tumor progression caused by the GSTM3 genotype [13]. Indeed, Lichy et al found a marked decrease in YY1 binding in malignant HeLa/fibroblast somatic cell hybrids when compared to non-tumor cells, [14] while Austen and colleagues showed that YY1 is a negative regulator of cell growth via potent inhibition of c-myc transforming activity and possible involvement in tumor suppression [15]. These diverse YY1 functions probably result from its ability to interact with numerous proteins and complexes.

YY1 is the only known mammalian Polycomb Group (PcG) protein with DNA binding specificity. We found that YY1 is functionally similar to the apparently orthologous *Drosophila* PcG protein, Pleiohomeotic (PHO) [16]. YY1 can repress transcription in a PcG-dependent fashion, can recruit PcG proteins to DNA, can correct phenotypic defects in PHO mutant flies, and can control genes needed for development and differentiation [17], [18], [19], [20]. In mammals, PcG proteins are implicated in Homeobox (Hox) gene regulation and stable silencing of specific sets of genes through chromatin modifications. PcG proteins are also involved in maintenance of embryonic and adult stem cells. The PcG protein Bmi-1 is necessary for hematopoietic stem cell (HSC) self-renewal and can control cell proliferation [17], [21], [22], [23]. Similarly, the PcG protein EZH2 can prevent HSC exhaustion [22] whereas Mel18 negatively regulates HSC self-renewal [24]. As YY1 is a PcG protein, it may also play important roles in HSC biology, but such roles have never been explored.

B cell development involves progression from Lin^−^Sca-1^+^c-Kit^+^ (LSK) progenitor cells through common lymphoid progenitors, pro-B, pre-B, immature B, mature B, and plasma cell stages. Transcription factors regulate cell fate determination through a complex network required for development of early progenitors, and for B cell lineage commitment, maturation, proliferation and survival [8], [25], [26], [27], [28]. YY1 has long been believed to play an important role in immunoglobulin (Ig) gene regulation because it binds to numerous Ig enhancer elements including the Igκ 3′ enhancer, the heavy chain intron enhancer, and the heavy chain 3′ enhancer [29], [30]. The Shi laboratory showed that YY1 is also a critical regulator of B cell development [31], as conditional knock out of the *yy1* gene in the B cell lineage results in an early B cell defect with an almost complete block at the pro-B cell stage, impaired V_H_ to D_H_J_H_ recombination, and reduced Ig locus contraction [31], [32].

Since conditional *yy1* gene knock-out in the B cell lineage profoundly impacts B cell development, various dosages of YY1 impact gene expression and development, and multiple PcG proteins where previously demonstrated to play crucial roles for hematopoietic stem cell self-renewal, we sought to explore the impact of YY1 over-expression on hematopoietic development. We utilized a mouse bone marrow reconstitution system to explore whether elevated levels of YY1 would impact hematopoietic cell development and growth. We found that elevated YY1 expression resulted in a progressive selective disadvantage as cells matured to the pro-B, pre-B, immature B and re-circulating B cell stages, but had no detrimental effect on bone marrow myeloid lineage cells. On the contrary, we observed an enrichment of YY1-expressing cells in the Lineage^−^, Sca-1^hi^, c-Kit^hi^ cell fraction, containing HSCs. This enrichment was particularly visible within the long-term HSC (LT-HSC) compartment. YY1 over-expression in B cell lines inhibited survival by inducing apoptosis, whereas apoptosis was not observed in a myeloid line. Two anti-apoptotic genes, Bcl-xl and NFκB2, were down regulated by elevated levels of YY1 in B cell lines but not in a myeloid cell line confirming the pro-apoptotic effect of YY1 in the B cell lineage.

## Results

**Figure 1:**
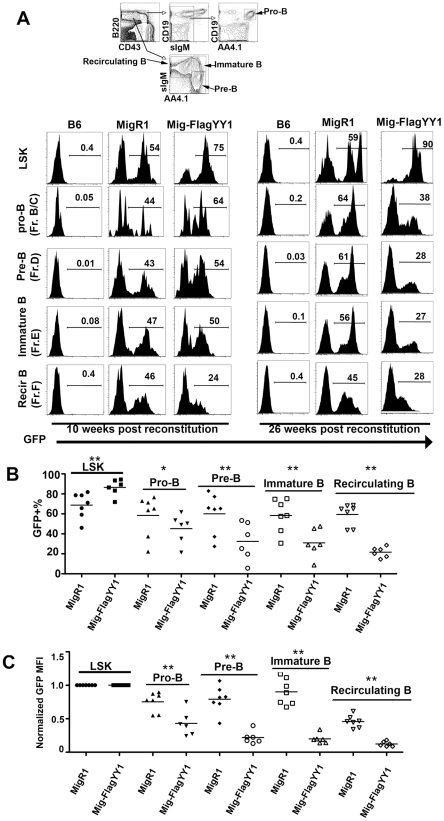
Cells expressing high levels of YY1 are depleted in B cell populations. Lethally irradiated C57BL/6 mice were reconstituted with 5-FU-treated C57BL/6 donor bone marrow cells transduced with MigR1-FlagYY1 or MigR1 vector alone. Recipient mice were subject to analysis at 10 weeks, 14 weeks, 18 weeks or 26 weeks post transplantation. (A) The top panel shows the cell gating strategy. Bone marrow cells were evaluated for the presence of pro-B (B220^+^CD43^+^CD19^+^AA4.1^+^IgM^−^), pre-B (B220^+^CD43^−^ IgM^−^AA4.1^+^), immature B (B220^+^CD43^−^IgM^+^AA4.1^+^), and re-circulating B (B220^+^CD43^−^IgM^+^AA4.1^−^) cells. GFP+ percentages are listed in each B cell compartment in MigR1 or MigR1-FlagYY1 reconstituted mice 10 weeks (left panel) or 26 weeks (right panel) post reconstitution. (B) MigR1-FlagYY1 reconstituted mice have lower GFP+ percentages compared with MigR1 control mice. GFP+ percentages shown for individual mice in the LSK, pro-B, pre-B, immature B and re-circulating B cell compartments at 14 or 18 weeks post reconstitution. Each data point represents a GFP+ percentage of an individual mouse. (C) GFP MFI decreases throughout B cell development in MigR1-FlagYY1 reconstituted mice. GFP MFI of bone marrow pro-B, pre-B, immature B and re-circulating B cells in MigR1-FlagYY1 and MigR1 reconstituted mice are normalized to GFP MFI in the LSK population of each group (MigR1 versus MigR1-FlagYY1). Each dot represents the MFI of an individual mouse 14 or 18 weeks post reconstitution. In (B) and (C), cell fractions marked with one or two asterisks show significant differences between MigRI and MigR1-FlagYY1 populations at P<0.05 and 0.01, respectively.

Conditional YY1 inactivation in the B cell lineage results in developmental arrest at the pro-B cell stage [31]. Haplo-insufficiency of YY1 can lead to numerous developmental defects and two-fold changes in YY1 expression can alter the regulation of many genes [4], [5]. As the impact of elevated YY1 expression on the B cell lineage is unknown, we set out to assess the effects of elevated YY1 expression on B cell development using a bone marrow transduction and reconstitution approach. For these studies, we transduced C57BL/6 bone marrow progenitors with retroviral vector alone (MigR1) or a retrovirus expressing Flag-tagged YY1 (MigRI-FlagYY1). The MigR1 vector allows us to track levels of YY1 expression by monitoring levels of green fluorescent protein (GFP) expression from a bicistronic RNA containing an internal ribosomal entry site. Thus, levels of GPF expression correlate with levels of YY1 expression when using this vector (Fig. S1A). In addition, we compared the level of YY1 expression in B lineage 38B9 pro-B cells and myeloid lineage 32D cells. Cells were transduced with MigR1-FlagYY1 vector and sorted into identical GFP low and GFP high gated fractions. Equivalent amounts of lysates were analyzed on the same gel by western blot with anti-YY1 antibody and anti-actin to serve as a loading control so we could directly compare YY1 levels in the two lineages. We found that myeloid cells expressed higher levels of endogenous YY1 compared to B lineage cells (Fig. S1B left, lanes 1 and 4). As expected, transduction with MigR1-FlagYY1 resulted in increased YY1 expression with the highest levels in the GPF high fractions (Fig. S1B lanes 2,3,5,6). Quantitation of these data are shown in Fig. 1B, right. Levels of total YY1 expression are overall similar in the two lineages, but are higher in the myeloid cells due to their higher level of endogenous YY1.

MigR1-Flag-YY1 or MigR1 transduced bone marrow cells were transplanted into lethally irradiated C57BL/6 mice. Reconstituted mice were subject to analysis from 10 weeks to 26 weeks post injection. Both MigR1 and MigR1-Flag-YY1 transduced bone marrow cells yielded efficient reconstitution efficiency at 10 weeks post injection as evidenced by the high percentage of GFP positive cells in the Lin^−^Sca^hi^ c-Kit^hi^ (LSK) fraction (Fig. 1A). We isolated GFP+ lymphoid cells by FACS from the blood of MigR1-FlagYY1 reconstituted mice, and performed western blots to determine the level of exogenous YY1 protein compared to endogenous YY1 protein. In blood lymphocytes, exogenous Flag-tagged YY1 protein (Fig. S1C left, upper band) was expressed at an equivalent level compared with endogenous YY1 (Fig. S1C left, lower band). Exogenous expression levels were similar to levels observed in transduced 32D myeloid cells (Fig. S1C, right). These experiments (Figure S1) show that cells expressing MigR1-FlagYY1 reconstitute well in recipient mice, that expression of YY1 is elevated in MigRI-FlagYY1 transduced cells, that myeloid and B lineage cells express similar levels of transduced YY1, and that GFP levels correlate with YY1 expression levels.

### YY1 over-expression results in a B lymphoid-specific selective disadvantage

Between 10 to 26 weeks post bone marrow reconstitution, recipient mouse bone marrow and spleen cells were stained and analyzed by flow cytometry. For MigR1 reconstituted mice, the percentage of GFP positive cells remained stable when comparing bone marrow pro-B (B220^+^CD43^+^CD19^+^AA4.1^+^ IgM^−^), pre-B (B220^+^CD43^−^ IgM^−^AA4.1^+^), immature B (B220^+^CD43^−^IgM^+^AA4.1^+^) and re-circulating B cells (B220^+^CD43^−^IgM^+^AA4.1^−^) (Fig. 1A, left panel). However, as early as 10 weeks post reconstitution, in MigR1-FlagYY1 reconstituted mice, there was a progressive decrease in percentage of GFP positive cells from 75% in LSK (Lin^−^Sca^hi^c-Kit ^hi^) cells, to 64% in pro-B, 54% in pre-B, 50% in immature B, and 24% in re-circulating B cells, respectively (Figure 1A, left panel, and Fig. 1B). In addition, there was a significant and progressive leftward shift of GFP signal in pro-B, pre-B, immature B, and re-circulating B cell pools indicating a selection against high level YY1 expression in the B cell lineage (Fig. 1A, left panel, and Fig. 1C). Consistent with less pronounced effects of YY1 expression in early B lineage cells, there was no noticeable difference in VDJ rearrangement of proximal and distal V_H_ genes in MigR1-FlagYY1 transduced animals compared to MigR1 vector alone (Fig. S2).

At 26 weeks post reconstitution, there continued to be a pronounced selection against cells expressing high levels of YY1 in the B cell lineage as evidenced by an even more obvious decrease of GFP percentages as well as leftward shifts of GFP signals (Fig. 1A, right panel). MigR1-FlagYY1 reconstituted mice had consistently lower percentages of GFP+ cells in bone marrow pro-B, pre-B, immature B and re-circulating B cells compared with MigR1 reconstituted mice (Fig. 1B). In addition, YY1 reconstituted mice had a substantially lower GFP mean fluorescence intensity (MFI) among GFP+ bone marrow pro-B, pre-B, immature B and re-circulating B lineages as compared with MigR1 reconstituted mice (Fig. 1C). This provided quantitative evidence for the loss of high YY1-expressing cells in these cell compartments. Thus high levels of YY1 expression appear to be detrimental to developing bone marrow B cell populations.

**Figure 2:**
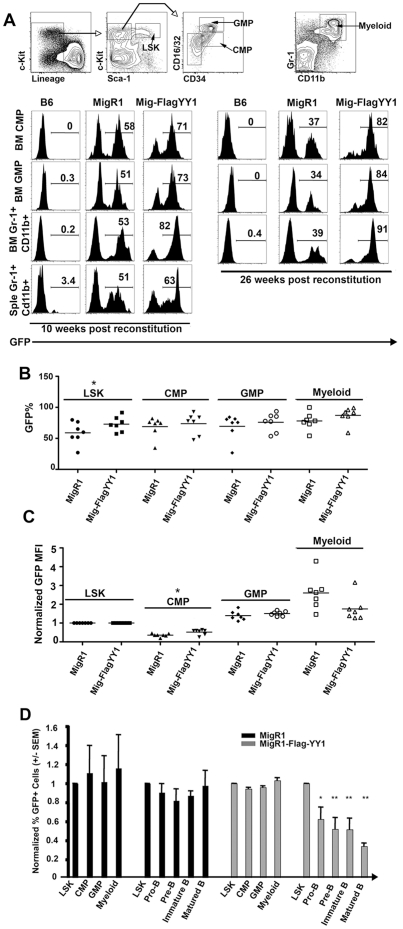
High levels of YY1 do not affect myeloid lineage development. Chimeric mice were prepared as described in Fig. 1. Recipient mice were subject to analysis at 10 weeks, 14 weeks, 18 weeks or 26 weeks post transplantation. (A) The top panel shows the cell gating strategy. Bone marrow cells were evaluated for the presence of CMP (lin^−^ c-kit ^hi^ Sca^−^ CD16/32^lo^ CD34^+^), GMP (lin^−^ c-kit ^hi^ Sca^−^ CD16/32^hi^ CD34^+^), and myeloid (Gr-1^+^CD11b^+^) cells. GFP+ percentages are listed in each cell compartment in MigR1 or MigR1-FlagYY1 reconstituted mice 10 weeks (left panel) or 26 weeks (right panel) post reconstitution. (B) GFP+ percentages of MigR1-FlagYY1 reconstituted mice are the same as MigR1 control mice in myeloid lineage cells. GFP+ percentages are shown for individual mice in the LSK, CMP, GM and myeloid cells in reconstituted mice 14 or 18 weeks post reconstitution. Each data point represents a GFP+ percentage of an individual mouse. (C) GFP MFI remains stable throughout myeloid cell development in MigR1-FlagYY1 reconstituted mice. GFP MFI of bone marrow LSK, CMP, GMP and myeloid cells in MigR1-FlagYY1 and MigR1 reconstituted mice are normalized to GFP MFI in the LSK population of each group (MigR1 versus MigR1-FlagYY1). Each dot represents the MFI of an individual mouse 14 or 18 weeks post reconstitution. (D) Cells expressing high levels of YY1 experience negative selection in the B cell lineage but not the myeloid lineage. GFP+ percentages of CMP, GMP, myeloid, pro-B, pre-B, immature B and re-circulating B cells are normalized to the GFP+ percentage of bone marrow LSK cells. Mean and standard error of the mean are shown. In B, C, and D, cell fractions marked with one or two asterisks show significant differences between MigRI and MigR1-FlagYY1 populations at P<0.05 and 0.01, respectively.

The YY1 inhibitory effect observed during B cell development was not observed in bone marrow myeloid lineages. At 10 weeks post reconstitution, the percentage of GFP positive cells remained relatively stable in the myeloid lineage from 75% in the LSK (Lin^−^Sca^hi^c-Kit ^hi^) pool (Fig. 1A) to 71% in bone marrow CMP (Lin^−^ Sca^−^ c-Kit ^hi^ CD16/32^lo^ CD34^+^), 73% in bone marrow GMP (lin^−^ c-kit ^hi^ Sca^−^ CD16/32^hi^ CD34^+^), and 82% of bone marrow myeloid cells (Gr-1^+^CD11b^+^) (Fig. 2A, left panel). GFP expression in splenic Gr-1^+^CD11b^+^ cells also remained relatively high at 63% (Fig. 2A, left panel). Similarly, at 26 weeks post reconstitution, we did not observe any detrimental effects of high levels of YY1 on bone marrow myeloid lineages (Fig. 2A, right panel). Compared to 10 weeks post-reconstitution, bone marrow CMP GFP positive cells increased from 71% to 82%, GMP increased from 73 to 84%, and Gr-1^+^CD11b^+^ cells increased from 82% to 91% (Fig. 2A). MigR1-Flag YY1 and MigR1 vector reconstituted mice had similar percentages of GFP positive cells in CMP, GMP and myeloid compartments (Fig. 2B), and MigR1-FlagYY1 and MigR1 vector reconstituted LSK, GMP and myeloid cells showed similar MFI among GFP+ cells, although there was a slight positive effect of YY1 on MFI in CMPs (Fig. 2C). The distinct effects of YY1 expression on the percentage of GFP+ cells among B cell and myeloid cell populations is summarized in Fig. 2D.

### Cells expressing elevated YY1 are enriched in LT-HSC cells

**Figure 3:**
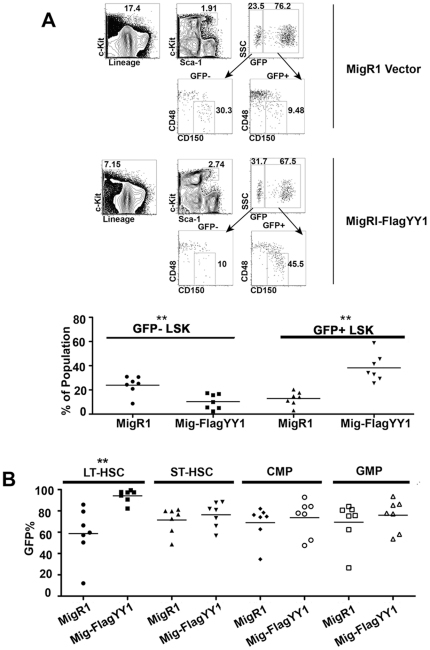
Cells expressing high levels of YY1 are enriched in the LT-HSC cell population. Chimeric mice were made as described in Figs. 1 and 2. (A) MigR1-FlagYY1 reconstituted mice show a relative enrichment in the LSK fraction. MigRI and MigR1-FlagYY1 reconstituted bone marrow cells were sorted into GFP− and GFP+ populations and the percentage of LSK cells in each fraction are plotted. (B) YY1 expressing cells are enriched in the LT-HSC fraction but not in the ST-HSC, CMP, or GMP fractions. In (A) and (B), cell fractions marked with two asterisks show significant differences between MigRI and MigR1-FlagYY1 populations at P<0.01.

PcG proteins are involved in hematopoietic development, stem cell self-renewal and cell proliferation. As YY1 is a PcG protein [17], we hypothesized that YY1 over-expression might also influence HSC biology. Consistent with this hypothesis, we observed an enrichment of MigRI-FlagYY1 transduced cells in the LSK fraction containing HSCs (Figure 1B). At 10 weeks post reconstitution, 75% of cells in the LSK pool were GFP positive and this increased to 90% by 26 weeks, indicating enrichment of cells expressing high levels of YY1 in this progenitor compartment (Fig. 1A). Within the LSK pool we further examined the phenotypically defined LT-HSC population by analysis of CD48 and CD150 cell surface markers [33], By comparing the percentages of LT-HSCs among GFP+ versus GFP− LSK cells, we observed a higher percentage of LT-HSCs within the GFP+ LSK population of YY1 reconstituted mice as compared to the GFP− population (Fig. 3A). Conversely, we examined the percentage of GFP expression in phenotypically defined LT-HSCs, short-term HSCs, common myeloid progenitors (CMPs), and granulocyte-macrophage progenitors (GMPs). An enrichment for GFP+ cells was observed specifically in the LT-HSC compartment of MigR1-FlagYY1 reconstituted mice but there was no difference in CMP and GMP populations (Fig. 3B). These data indicate that cells expressing high levels of YY1 are enriched in the bone marrow LT-HSC population of MigR1-FlagYY1 reconstituted mice.

### YY1 inhibits B cell growth in vitro

**Figure 4:**
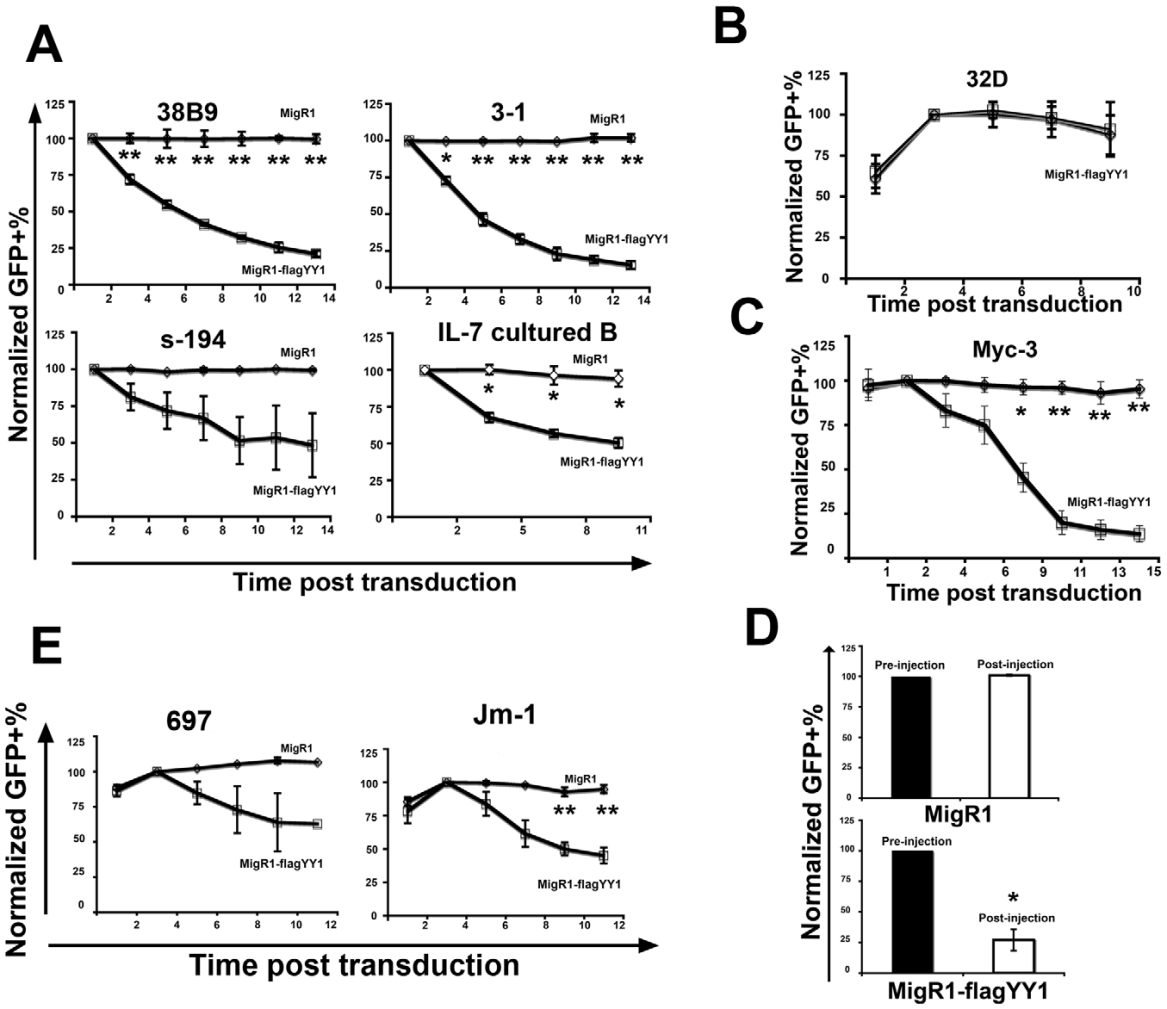
Transduction with MigR1-FlagYY1 results in selective disadvantage in the B cell lineage in vitro and in vivo, but not in myeloid cells. (A) Murine pro-B cell line 38B9, pre-B cell line 3-1, plasmacytoma cell line S194 and Il-7 cultured murine bone marrow cells were retrovirally infected with MigR1-FlagYY1 or MigR1 empty vector. Percentage of GFP positive cells was checked every two days by flow cytometry. Percentages of GFP positive cells were normalized to the level at 48 hours post-transduction. (B) Murine myeloid cell line 32D was transduced by MigR1-FlagYY1 or MigR1 retrovirus vectors. Percentage of GFP positive cells was checked every other day by flow cytometry for 10 days. Percentages of GFP positive cells were normalized to the level on day 4 post-transduction. (C) Myc3 B-lymphoma cells were transduced with MigR1-YY1 or MigR1 control vector. Percentage of GFP positive cells was checked every two days by flow cytometry. Percentages of GFP positive cells were normalized to the level on day 2 post-transduction. (D) Two days post infection, GFP+ percentages of Myc-3 cells were monitored by flow cytometry (pre-injection) and transduced Myc3 cells were then injected into syngeneic mice subcutaneously. The number of GFP positive Myc3 cells in each sample was then analyzed after tumor development two weeks post-injection. Percentages of GFP positive cells were normalized to the level on day 2 post-transduction. *E*, Human pre-B ALL cell lines 697 and JM-1 were transduced by MigR1-YY1 and MigR1 retrovirus vectors. Percentages of GFP positive cells were normalized to levels on day 4 post-transduction. The data represent the mean and standard error of the mean from three independent experiments. In each panel, cell fractions marked with one or two asterisks show significant differences between MigRI and MigR1-FlagYY1 populations at P<0.05 and 0.01, respectively.

To further explore the inhibitory activity of YY1 expression in the B cell lineage, we tested the effect of YY1 expression on the growth of various B cell lines. We reasoned that if YY1 inhibited B cell growth, cells transduced with MigR1-FlagYY1 should be at a selective growth disadvantage in culture and should progressively decrease in relative percentage compared to untransduced cells and vector control transduced cells. We retrovirally transduced murine pro-B cell line 38B9, murine pre-B cell line 3-1, murine plasmacytoma cell line S194, and Il-7 cultured primary bone marrow B cells with MigR1-FlagYY1 or empty MigR1 vector. MigR1 vector transduced GFP-positive cells proliferated at a rate similar to the untransduced GFP-negative cells as indicated by the stable percentage of GFP-positive cells at different time points after transduction (Fig. 4A). In contrast, the percentage of MigR1-YY1 transduced GFP positive cells declined dramatically in murine pro-B, pre-B, plasmacytoma, and Il-7 cultured bone marrow B cells, indicating growth arrest and/or cell death (Fig. 4A). Since our in vivo data showed that YY1 overexpression was detrimental to B cells but not myeloid cells (Figs. 1 and 2), we transduced murine myeloid cell line 32D with MigR1-YY1 or MigR1 empty vector. Consistent with our in vivo data, there was no decline of GFP-positive percentages in YY1 expressing myeloid cells over time (Fig. 4B). Thus, similar to the case in vivo, we observed no negative impact of elevated YY1 levels on myeloid cell growth.

To test the impact of elevated YY1 on B-lymphomagenesis in vivo, the Myc-over-expressing B-lymphoma Myc3 [34] was infected with either MigR1-YY1 or MigR1 vector. When evaluated in vitro, YY1 transduced GFP positive cells declined dramatically over time (Fig. 4C). To test YY1 inhibitory effects in vivo, the mixture of transduced and uninfected cells was injected subcutaneously in syngeneic animals to produce tumors. Two weeks post-injection, the resulting tumors were analyzed by flow cytometry and percentages of GFP-positive cells were determined again. For MigR1 infected cells, the percentage of GFP-positive cells remained unchanged (Fig. 4D). However, there was a 3-fold decrease in the percentage of GFP-positive cells in MigR1-FlagYY1 transduced Myc3 cells (Fig. 4D). We also tested two human pre-B acute lymphoblastic leukemia (ALL) lines (697 and JM-1) to determine whether they would also respond to elevated YY1 expression similar to murine B cell lines. The two cell lines were transduced with MigR1 or MigR1-FlagYY1 and the percentage of GFP-positive cells was plotted over 10 days. Again, there was a drop in GFP-positive cells over time in the YY1-elevated samples, similar to the case with the murine B lymphoma cell lines (Fig. 4E). Thus, in all cases, YY1 expression resulted in a disadvantage among B lineage cells, but had no impact on myeloid cells.

### YY1 can induce apoptosis in B cells

**Figure 5:**
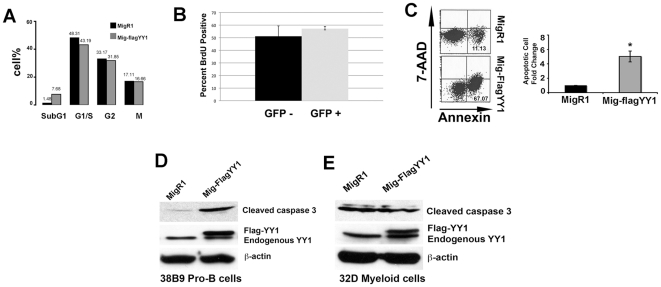
High levels of YY1 induce apoptosis in B cells but not in myeloid cells. (A) MigR1-FlagYY1 and MigR1 transduced 38B9 cells were stained with propidium iodide and the percentage of cells at each phase of the cell cycle were determined by flow cytometry. *C*, Cleaved caspase 3 is up regulated in MigR1-FlagYY1 transduced pro-B cell line. MigR1-YY1 and MigR1 transduced 38B9 cell lysates were immunoblotted for cleaved caspase 3 and YY1. β-actin was used as a loading control. (B) YY1 overexpression does not change the percentage of cycling cells. 38B9 cells were transduced with MigR1-YY1, sorted by FACS into GFP+ and GFP− populations and incubated with Brdu to measure DNA synthesis. (C) Murine pro-B cell line 38B9 was infected with MigR1-YY1 or MigR1 vectors such that 90% of cells were transduced. Cells were stained with Annexin V-APC and 7-AAD, and early apoptotic cells (Annexin-V+ and 7-AAD−) were determined by flow cytometry 48 hours post infection. Percentages of apoptotic cells were normalized to percentage of apoptotic cells of MigR1 vector only infected cells. Mean and standard error of the mean are shown. The asterisk indicates significant differences between MigRI and MigR1-FlagYY1 populations at P<0.05. (D) Cleaved caspase 3 is not up regulated in a MigR1-FlagYY1 transduced myeloid cell line. Murine myeloid cell line 32D was transduced with MigR1-FlagYY1 or MigR1 vectors and GFP positive cells were isolated by FACS. Cell lysates made from sorted cells were immunoblotted for cleaved caspase 3 and YY1. β-actin was used as a loading control. The upper band indicates the Flag-tagged exogenous YY1 and the lower band is endogenous YY1.

To determine the mechanism of YY1-induced selective disadvantage in B cells, we assessed whether elevated YY1 expression affected cell growth parameters. MigR1 and MigR1-YY1 transduced 38B9 cells were evaluated for DNA content by PI staining. Comparing MigR1-FlagYY1 to empty vector there was little difference in G1/S, G2, or M stage profiles (Fig. 5A). Similarly, there was no difference in Budr incorporation comparing GFP+ and GFP− 38B9 cells transduced with MigRI-YY1 (Fig. 5B). However, we observed a 7 fold increase in the sub-G1 phase cell population consistent with a substantial increase in apoptotic cells (Fig. 5A). MigR1-FlagYY1 and MigR1 transduced 38B9 pro-B cells were stained with the dead cell exclusion dye 7-AAD and the apoptosis indicator AnnexinV. Interestingly, we observed a substantial increase of early apoptotic cells (AnnexinV^+^, 7AAD^−^) in MigR1-FlagYY1 transduced cells as compared to those transduced with MigR1 empty vector (Fig. 5C).

We also tested the impact of elevated YY1 on the apoptosis marker protein, cleaved caspase-3. In MigR1-FlagYY1 transduced 38B9 pro-B cells, increased YY1 expression led to a 6.6 fold elevation of cleaved caspase-3 (Fig. 5D). As a negative control, we transduced the murine myeloid cell line 32D with MigR1-FlagYY1 or MigR1 and GFP-positive cells were sort-purified. Increased YY1 expression did not change the level of cleaved caspase-3 when comparing MigR1-FlagYY1 expressing cells to empty vector MigR1 expressing cells (Fig. 5E). Thus, consistent with our in vivo results, forced expression of YY1 did not cause apoptosis in myeloid cells.

**Figure 6:**
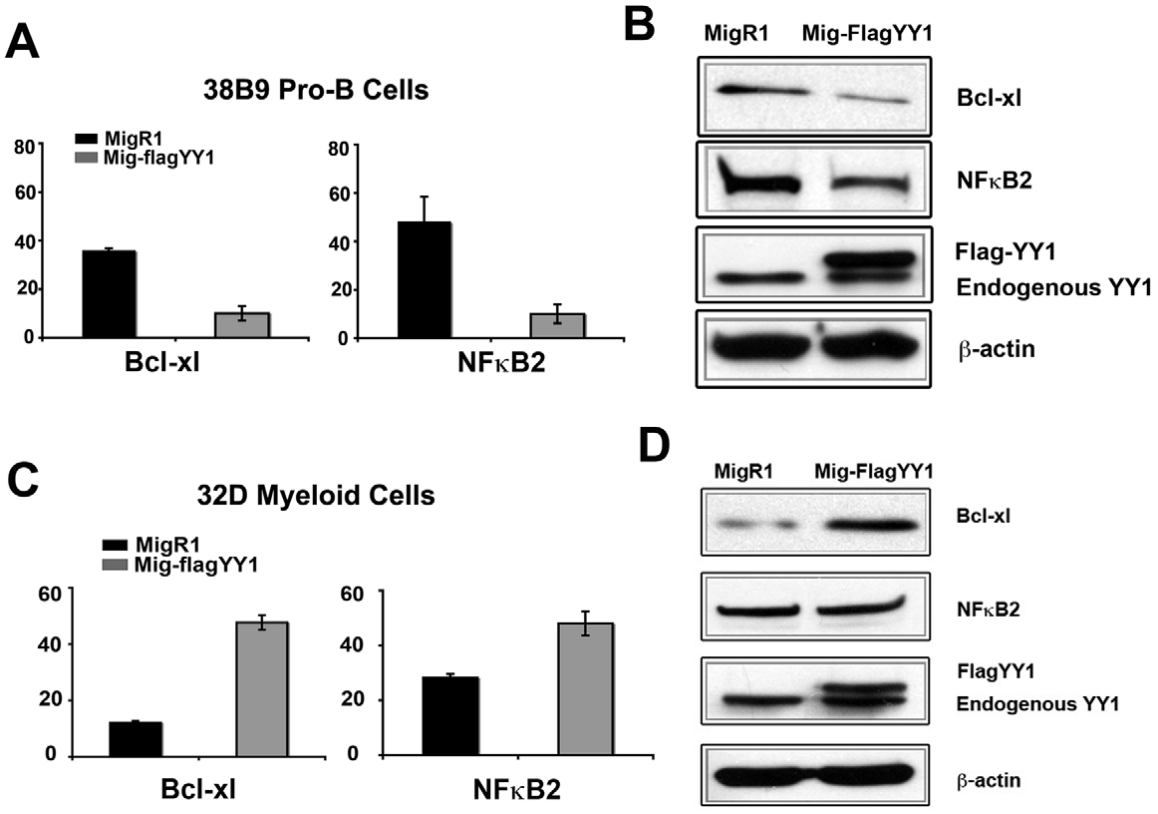
Anti-apoptotic genes NFκB2 and Bcl-xl are down regulated by YY1 in murine pro-B cells but not in murine myeloid cells. (A) NFκB2 and Bcl-xl are down regulated at the transcript level in murine 38B9 pro-B cells. RT-PCR analyses of NFκB2 and Bcl-xl mRNA levels relative to hprt are plotted for MigR1-YY1 or MigR1 transduced 38B9 cells. The data represent the mean and standard error of the mean for three independent experiments. (B) NFκB2 and Bcl-xl are down regulated by YY1 at the protein level in murine 38B9 pro-B cells. Cell lysates from MigR1-YY1 or MigR1 transduced 38B9 cells were immunoblotted for Bcl-xl, NFκB2 and YY1. β-actin was used as a loading control. (C) NFκB2 and Bcl-xl are up regulated at the transcript level in murine myeloid cell line 32D. GFP positive MigR1-YY1 and MigR1 transduced murine myeloid cell line 32D samples were isolated by FACS. Levels of NF-kB2 and Bcl-xl transcripts were determined by RT-PCR and hprt was used as an internal control and for normalization. The data represent the mean and standard error of the mean from three independent experiments. (D) GFP positive MigR1-YY1 and MigR1 transduced murine myeloid cell line 32D samples were isolated by FACS. Cell lysates were made from sorted cells and immunoblotted for NFκB2, Bcl-xl and YY1. β-actin was used as a loading control. Mean and standard error of the mean are shown. Asterisks indicate significant differences between samples at P<0.05.

To gain further insight into YY1 induced B cell-specific apoptosis, we used a genome-wide microarray approach to identify YY1 target genes in murine pro-B cells. We performed microarray analyses on 38B9 pro-B cells transduced either with MigR1-FlagYY1 or MigR1 vector control. This comparison enabled us to identify approximately 700 genes whose expression was affected by YY1. RT-PCR analyses for a subset of transcripts confirmed our microarray results as over 80% of transcripts matched closely in fold increase or decrease in the presence of overexpressed YY1 (Fig. S3; see also Fig. 6A).

Evaluation of identified transcripts using David Bioinformatics Resources (NIAID) and the KEGG Functional Annotation Chart revealed that genes involved in cell cycle control and BCR-ABL signaling were down-regulated by YY1. The complete list of YY1-responsive genes is provided in Table S3. Consistent with the YY1-induced apoptotic effect we observed in B cells, several anti-apoptotic related genes (NFκB2 and Bcl-xl) were deregulated by elevated YY1 expression. We found 4 fold decreases in NFκB2 and Bcl-xl transcripts in MigR1-FlagYY1 transduced cells compared to MigR1 vector alone (Fig. 6A) and a corresponding 2.4 fold decrease in the levels of NFκB2 and 3.4 fold decrease in Bcl-xl proteins (Fig. 6B). On the contrary, in MigR1-FlagYY1 transduced 32D myeloid cells we observed the opposite results. In contrast to YY1-induced down regulation of Bcl-xl and NFκB2 in B cells, we observed a 4.5-fold increase in Bcl-xl transcripts and 2.6-fold increase in Bcl-xl protein in 32D myeloid cells. Similarly, we observed a 1.5-fold increase of NFκB2 transcripts but no change of NFκB2 protein in 32D myeloid cells (Fig. 6C and D). Thus, elevated YY1 results in down regulation of anti-apoptotic genes in B cells but not in myeloid cells.

The mechanism of down regulation of NFκB2 and Bcl-xl by YY1 in B cells could be either direct or indirect. ChIP assays were performed to determine if YY1 bound directly to the Bcl-xl and NFκB2 promoters. Multiple primer sets were designed covering approximately 1 kb of the Bcl-xl and NFκB2 promoters, respectively. Whereas YY1 was clearly enriched at the positive control rpL30 promoter in 38B9 pro-B cells, we did not detect obvious enrichment of YY1 at either Bcl-xl or NFκB2 promoters above that observed at the negative control b-actin promoter (Figure S4). Thus, YY1 may influence these two anti-apoptotic genes indirectly, or YY1 might bind to other DNA sequences that regulate these two genes. Consistent with the former option, overexpression of NFkB2 and Bcl-xl either separately or together failed to reverse the pro-apoptotic effect of YY1 overexpression (data not shown). Thus, reduced NFkB2 and Bcl-xl proteins in YY1 overexpressing B cells appears to be a consequence of YY1 apoptotic induction rather than due to direct regulation of the NFkB2 and Bcl-xl genes. None-the-less, reduced levels of these proteins confirm that YY1 overexpression induces apoptosis in B lineage cells.

## Discussion

The functional consequences of YY1 regulation in hematopoietic cell development are poorly understood. Conditional knock-out of YY1 in the B cell lineage by action of *mb1*-driven CRE results in arrest at the pro-B cell stage and impairment of immunoglobulin distal V gene rearrangement [31]. Loss of YY1 may have an impact on both immunoglobulin locus contraction needed for rearrangement of the most distal V genes, as well on cell proliferation [31], [32]. Other investigators have shown that YY1 can inhibit neutrophil differentiation both in vivo and in vitro [35]. In this study, we used bone marrow transduction and reconstitution to investigate the impact of elevated YY1 expression on the B cell and myeloid hematopoietic compartments in vivo. Significantly, we found that forced expression of YY1 resulted in selective disadvantage as cells developed within the B cell lineage. We observed a progressive decline in the percentage of YY1-transduced (GFP positive) cells at each successive B cell stage, and the GFP MFI signal progressively decreased indicating selection against cells expressing high levels of YY1 (Figs. 1 and 2). The inhibitory effect of YY1 on B cell development was not caused by a non-specific toxic effect of retroviral over-expression as bone marrow myeloid lineage cells developed normally (Fig. 2). In vitro studies confirmed the in vivo results showing selective disadvantage of high YY1 expression in B lineage cell lines, but not in myeloid cell line 32D. Thus, while YY1 over-expression leads to growth disadvantage in B lineage cells, there is no negative impact on myeloid cells. Our study is the first to show that YY1 over-expression negatively impacts B cell development.

It is interesting that loss of YY1 [31] and overexpression of YY1 (our results here) both result in altered B cell development. This situation is somewhat reminiscent of the impact of overexpression verses knock-out of transcription factor GATA-3. GATA-3 is essential for T cell development and knock-out severely impacts hematopoiesis [36], [37], [38]. However, overexpression of GATA-3 also blocks lymphoid and T lineage development [39], [40]. Thus, YY1 yields a similar scenario in which both loss of, and overexpression of YY1 impacts B cell development. However, the phenotypes of these two experimental systems are quite different. YY1 conditional knock-out results in arrested B cell development at the pro-B cell stage and reduced distal V_H_ gene rearrangement. On the contrary, cells overexpressing YY1 in the B cell lineage progress past the pro-B cell stage and show normal V_H_ gene rearrangement. However, YY1 overexpressing cells show reduced numbers of B cells at later stages of B cell development and show a progressive selection against cells expressing high levels of YY1. The reduction is most significant past the pro-B cell stage. Our microarray data indicate that YY1 overexpression impacts apoptosis related genes as well as growth control genes. Perhaps most relevant is the ability of YY1 to inhibit expression of cyclin D3. This cyclin is very important for early B cell development and particularly for the proliferative expansion of cells between the pro-B and pre-B cell stages [41]. Knock-out of cyclin D3 causes a significant drop in cells progressing to the pre-B cell stage resulting in reduced cell numbers past the pro-B cell stage [41]. This is similar to the drop we observe in our YY1 overexpression studies. Thus, YY1 may inhibit cyclin D3 expression leading to reduction in the proliferative burst and reduced differentiation to the pre-B cell stage.

In addition, cyclin D3 is required for germinal center development and expansion of late stage germinal center B cells [42], [43]. Interestingly, cyclin D3 knock-out does not impact cell proliferation in vitro, but results in failed proliferative expansion of late stage germinal center B cells [42], [43]. Thus, the impact of YY1 overexpression on reduced B cell numbers past the pro-B cell stage may at least partially relate to inhibition of cyclin D3 which is exquisitely important for two proliferative expressions in the B cell lineage.

Others have also shown that modulation of YY1 expression leads to altered expression of genes involved in cell cycle regulation, mitosis and cytokinesis, DNA replication, apoptosis, and cell growth [5]. For instance, complete ablation or knock-down of YY1 leads to growth inhibition in embryonic fibroblasts, HeLa cells, DT40 cells, and embryonic carcinoma cells, [5], [12], [44]. However, YY1 expression can similarly regulate cell growth by interaction with Rb [8], [12], [44]. YY1 can also impact senescence by regulating p16^INK4a^ [45], and can increase HoxB7 expression directly involved in tumor progression [46].

The negative impact of YY1 on B cell development could also be due to disrupted stoichiometry of YY1 in multiple protein complexes such as the PcG and INO-80 complexes, or due to interactions with regulatory proteins such as p53, c-myc, histone acetyltransferases, and histone deacetylases [2], [12], [17], [47]. Thus, over-expression of YY1 could influence protein complex formation leading to alterations in cell function. The negative impact of elevated YY1 levels on B cell development could also be the consequence of growth inhibition. However, our in vitro data did not reveal changes in cell cycle progression. Instead, a fraction of cells underwent apoptosis. Our in vitro results are most consistent with YY1 expression inducing increased apoptosis in B cells, but not in myeloid cells. Our study is the first to show that YY1 has a pro-apoptotic function in B cells.

In vivo, additional YY1 effects on stem cells and lineage differentiation are likely to be important. Unlike the case in B cells, YY1 expression resulted in enrichment of phenotypically defined LT-HSCs and granulocyte lineage cells. By 26 weeks post reconstitution, cells expressing high levels of YY1 constituted 90% of the LSK population and 91% of the bone marrow Gr-1+ population. The percentage of YY1-expressing reconstituted LT-HSCs and myeloid cells was substantially higher compared with MigR1 vector reconstituted LT-HSC indicating a positive selection for high levels of YY1 expression. These are interesting results because they suggest that elevated levels of YY1 expression might be beneficial for HSC function and may enhance differentiation to the myeloid lineage at expense of the B lymphoid lineage. This is reminiscent of a similar effect by transcription factor PU.1 [48].

Epigenetic states are implicated in controlling various HSC populations such as LT-HSC and ST-HSC, and YY1 expression may function to stabilize the LT-HSC chromatin phenotype. Alternatively, YY1 might function as a traditional transcriptional activator or repressor to regulate specific genes involved in cell cycle control and stem cell function. In preliminary studies we found that YY1 expression correlated with elevated c-kit expression in LSK cells (unpublished data). Thus, YY1 might regulate genes involved in maintenance of the HSC phenotype and self-renewal. Clinical strategies to enhance YY1 expression could thus preserve, or even enhance, hematopoietic reconstitution.

YY1 is a PcG protein and these factors function in the stable silencing of specific gene sets through chromatin modifications and dynamic reprogramming of epigenetic states. Many PcG proteins including Bmi-1, EZH2, Rae28, and Mel18 are involved in HSC function [22], [23], and forced expression of PcG protein EZH2 can prevent HSC exhaustion [22]. YY1 is the only mammalian PcG protein with specific DNA binding ability, and YY1 can recruit PcG proteins to specific DNA sites causing subsequent trimethylation of histone H3 on lysine 27 [18], [20]. Thus, forced expression of YY1 might change gene expression profiles by altering global chromatin structure.

Dysfunction of PcG proteins has been argued to contribute to the generation of cancer stem cells implying that PcG proteins provide potential targets for anticancer therapy [49], [50], [51]. For instance, loss or knock-down of PcG protein Ezh2 causes loss of cell growth and the transformed phenotype of prostate cancer cells [52]. Thus, the enrichment of cells expressing high levels of YY1 in the LT-HSC cell population argue that YY1 might be involved in cancer stem cell function and therefore could be a potential therapeutic target for some hematopoietic malignancies. The finding that transcription factor YY1 differentially controls hematopoietic development provides rationale for multiple future studies on the effect of YY1 on stem cell biology, lineage development, and growth control.

## Materials and Methods

All work involving animals complied with all federal, state, and local laws, and was done with approval and oversight of the University of Pennsylvania Institutional Animal Care and Use Committee under protocol 803080.

### Retroviral constructs

The flag-tagged YY1 cDNA sequence encoding the complete 414 amino acid protein was cloned into the HpaI site of GFP-expressing MSCV-IRES-GFP vector (MigR1) by blunt end ligation. High titer retroviral supernatants were prepared following transient transfection of HEK293 cells. Retroviral envelopes with ecotropic host ranges (pHIT123, for murine cells and pHIT 456 for human cells) were used.

### Bone marrow transduction and transplantation

Bone marrow cells were harvested from 6 to 8 week-old C57BL/6 or mb1-CRE yy1^f/f^ mice 4 days after intravenous injection of 250 mg/kg 5-fluorouracil (5-FU). Cells were cultured overnight in DMEM plus 10% fetal bovine serum and L-Glutamine, with IL-3 (6 ng/ml), IL-6 (5 ng/ml), and SCF (100 ng/ml), then washed and resuspended in retroviral supernatant containing polybrene (4 µg/ml) and the same cytokine cocktail for spin infection at 1,290 g for 90 minutes. A second round of spin infection was performed 24 hours following the first one. At least 5×10^5^ cells were injected intravenously into lethally irradiated (9 Gy) recipient C57BL/6 mice. Antibiotic containing drinking water was provided for recipient mice for two weeks post transplantation.

### Cell cultures

HEK293 cells [53] were maintained in DMEM with 10% FBS. Murine pro-B-cell line 38B9 and pre-B-cell line 3-1 [54] were cultured in RPMI 1640 supplemented with 10% fetal bovine serum (FBS) and 2-mercaptoethanol. Murine plasmacytoma cell line S194 (American Type Culture Collection) was cultured in DMEM with 10% horse serum. Myc3 B lymphoma cells [55] were cultured on a monolayer of gamma-irradiated S17 feeder cells [56] as described previously [55], [57]. Murine myeloid cell line 32D (American Type Culture Collection) was cultured in IMDM with 10% FBS, 10% WEHI-3B supernatant and antibiotics. Human pre-B ALL cell lines 697 [58] and JM-1 (American Type Culture Collection) were cultured in C10 lymphocyte media as previous described [59].

### Primary B cell cultures

Bone marrow cells from C57BL/6 mice were isolated, plated onto OP9 feeder cells and were cultured in IMDM with 10% FBS, 10 ng/mL IL-7, 50 micromolar 2-mercaptoethanol, 1× L-glutamine, 1× non-essential amino acids and 1× penicillin/streptomycin. Ten days later, 90% of bone marrow-derived cells were B220^+^IgM^−^CD43^+^ by flow cytometry.

### Antibodies and flow cytometry

Antibodies for FACS analyses were obtained from BD Biosciences-Pharmingen or eBioscience and are as follows: CD19 (1D3), c-kit (2B8), Sca-1/Ly-6A/E (E13-161.7), B220 (RA3-B2), Ter119, CD11b, Gr-1/Ly6G, CD16/32, CD34, CD43, AA4.1, sIgMb, CD21, CD 23, CD4, CD8 alpha, TCR beta, NK1.1, and CD25. Acquisition was performed on FACS Calibur FACSCanto or LSRII (BD). Sorting was performed with a FACSAriaII (BD). Data were analyzed with BD FACStation or FlowJo software.

### Western blots

Total cell extracts were prepared in 2× SDS sample buffer (Biorad). Sodium dodecyl sulfate-polyacrylamide gel electrophoresis, western blotting, and peroxidase-based chemiluminescence detection were performed according to standard laboratory protocols. Antibodies were purchased from Santa Cruz (YY1, H414), BD Biosciences (Bcl-xl, 2H12), or Cell Signaling (NFκB2, 4882; cleaved caspase-3, Asp175). Bands were quantified by using ImageJ software.

### PCR Detection of Ig Rearrangement

Genomic DNA from sorted GFP^+^ splenic B cells from MigR1 or YY1 reconstituted mice were made by DNeasy Blood & Tissue kit (QIAGEN). For heavy chain rearrangements, DNA samples were amplified by PCR reactions with primers described previously [60]. PCR products were separated on 1.5% agarose gels and visualized by Southern blot with an oligo probe (5-AGC CTT CAG GAC CAA GAT TCT CTG CAA ACG-3) detecting the region upstream of J_H_3.

### Quantitative real-time PCR

RNA was made by the Trizol procedure according to the manufacturer's specifications (Invitrogen) and subjected to the reverse transcriptase-polymerase chain reaction (RT-PCR) procedure. Murine primers for YY1 were: sense CCCACGGTCCCAGAGTCCA and anti sense TGTGCGCAAATTGAAGTCCAGT. Other primers for RT-PCR are listed in Table S1. Hprt was used as an internal control or normalization. Hprt primers were: sense CTCCTCAGACCGCTTTTTCC and antisense TAACCTCCTTCATCATCGCTAATC. All amplifications crossed intron-exon boundaries to exclude genomic DNA amplification.

### ChIP

Chromatin immunoprecipitation (ChIP) was carried out as previously described [61]. For all ChIP experiments, quantitative PCR analyses with primers shown in Table S2 were performed by real-time PCR using a Roche Lightcycler 1.5. Relative increases were calculated on the basis of pre-immune ChIP sample quantitative PCR values. Relative enrichments for each region are presented as the percentage of input.

### BrdU assay

BrdU was directly added into the culture medium to obtain a final concentration of 50 µM for 45 minutes. Cells were then fixed, permeabilized, and stained with APC conjugated anti-BrDU antibody following the manufacture's instructions (BrdU flow kit, BD Pharmingen). 7-AAD was added cells for FACS analyses.

### Microarray analyses

Murine pro-B 38B9 cells were retrovirally infected with MigR1 vector or MigR1-FlagYY1 such that nearly 100% of the cells were transduced. 48 hours post infection, RNA samples were prepared using Trizol reagent (Invitrogen), and cDNA was prepared using the Superscript II kit (Invitrogen). The cDNAs were subjected to hybridization with a DNA-Chip mouse genome 430A array (Affymetrix) using the University of Pennsylvania Microarray Facility's standard protocol (http://www.med.upenn.edu/microarr/DataAnalysis/Affymetrix/methods.htm). Data analyses were performed with Affymetrix Microarray Suite 5.0. The work complies with MIAME guidelines and has been deposited with ArrayExpress (accession number E-MTAB-679).

## Supporting Information

Figure S1 **GFP levels correlate with YY1 expression, and exogenous YY1 is well expressed in chimeric mice.** (A) 38B9 cells were tranduced with MigR1-FlagYY1 and four days later cells were sorted into low, middle, and high GFP-expressing populations. Cell lysates were evaluated by western blot with anti-YY1 antibody, and blots showed that YY1 expression correlates with the level of GFP expression. (B) 32D myeloid and 38B9 pro-B cells were transduced with MigR1-FlagYY1 and sorted into GFP low and GFP high fractions of identical intensity between the two cells types. Equivalent amounts of cell lysates from untransduced (endogenous) and GFP sorted samples were evaluated by western blot on the same gel with anti-YY1 antibody (414; Santa Cruz Biotechnology) or anti-actin antibody and signals were quantitated with Image J software. The left panel shows the western blot data from the same gel probed at the same time with the same antibodies. The right panel shows Image J quantitation. Endogenous YY1 levels are higher in myeloid cells and increase to slightly higher levels after MigRI-FlagYY1 transduction compared to 38B9 pro-B cells. (C) Exogenous YY1 is expressed at similar protein levels as endogenous YY1 in B cells. GFP+ lymphocytes were sorted from the blood of MigR1-FlagYY1 reconstituted mice 14 weeks post reconstitution and crude cell lysates were made. Western blot was performed for detection of both endogenous and exogenous Flag tagged YY1. The upper band indicates Flag-tagged exogenous YY1 and the lower band indicates endogenous YY1.
(TIF)

Figure S2 **VDJ rearrangements are similar in MigR1 vector and MigR1-FlagYY1 transduced B cells from reconstituted animals.** Rearrangement of various V_H_ gene families is shown comparing mice reconstituted with MigR1 vector alone or MigR1-FlagYY1. V gene and DJ rearrangements are not altered by YY1 expression.
(TIF)

Figure S3 **Confirmation of YY1 overexpression microarray results by RT-PCR.** Microarray results (black bars) shown as fold change increase or decrease of MigR1-FlagYY1 transduced 38B9 cells relative to MigR1 vector alone, are compared with fold changes measured by RT-PCR (stippled bars). Error bars show the standard deviation of the mean. All transcripts matched closely by the two methods except for nanog expression which was induced to a much lower level as determined by RT-PCR.
(TIF)

Figure S4 **YY1 does not bind at the Bcl-xl or NFκB2 promoter areas.** Chromatin made from murine 38B9 pro-B cells was immunoprecipitated with YY1 antibody or rabbit IgG control antibody. ChIP PCR was performed to detect the binding of YY1 at Bcl-xl or NFκB2 promoter areas. 8 sets of primers were designed to cover 1 kb of upstream promoter sequence of the Bcl-xl gene, and 6 sets of primers were designed to cover the NFκB2 promoter. RpL30 was used as a positive control for YY1 binding, and beta-actin was used as a negative control for YY1 binding. The mean and standard deviation are shown.
(TIF)

Table S1 **Real-Time PCR Primers.**
(DOCX)

Table S2 **Primers used for ChIP analyses.**
(DOC)

Table S3 **Microarray.**
(XLS)
