# Supplementary figures and images for: Increased Expression of PcG Protein YY1 Negatively Regulates B Cell Development while Allowing Accumulation of Myeloid Cells and LT-HSC Cells
Source: PLoS One. 2012 Jan 23;7(1):e30656. doi: 10.1371/journal.pone.0030656 (PMC3264595; doi:10.1371/journal.pone.0030656)

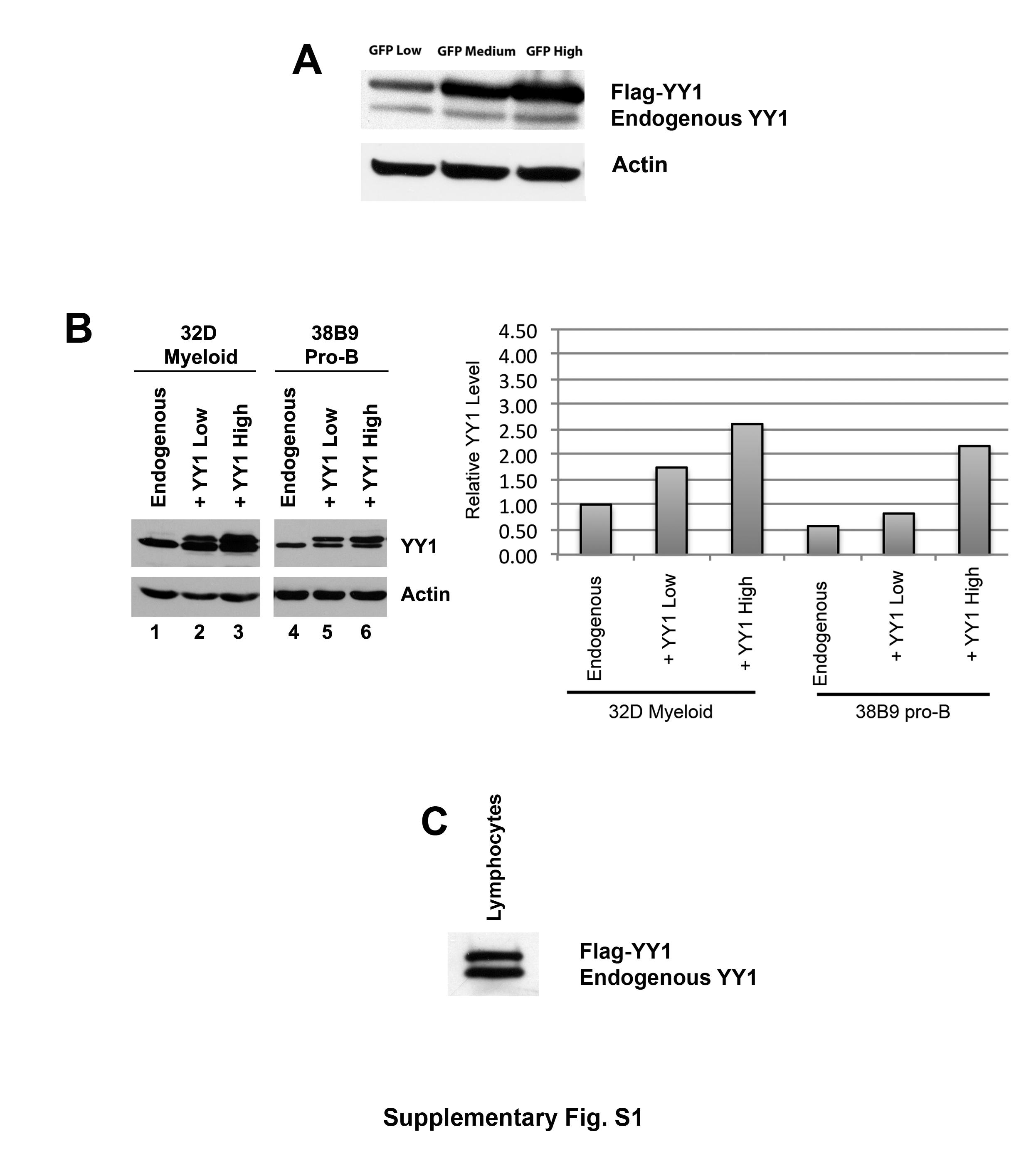

Supplement: Figure S1 — GFP levels correlate with YY1 expression, and exogenous YY1 is well expressed in chimeric mice. (A) 38B9 cells were tranduced with MigR1-FlagYY1 and four days later cells were sorted into low, middle, and high GFP-expressing populations. Cell lysates were evaluated by western blot with anti-YY1 antibody, and blots showed that YY1 expression correlates with the level of GFP expression. (B) 32D myeloid and 38B9 pro-B cells were transduced with MigR1-FlagYY1 and sorted into GFP low and GFP high fractions of identical intensity between the two cells types. Equivalent amounts of cell lysates from untransduced (endogenous) and GFP sorted samples were evaluated by western blot on the same gel with anti-YY1 antibody (414; Santa Cruz Biotechnology) or anti-actin antibody and signals were quantitated with Image J software. The left panel shows the western blot data from the same gel probed at the same time with the same antibodies. The right panel shows Image J quantitation. Endogenous YY1 levels are higher in myeloid cells and increase to slightly higher levels after MigRI-FlagYY1 transduction compared to 38B9 pro-B cells. (C) Exogenous YY1 is expressed at similar protein levels as endogenous YY1 in B cells. GFP+ lymphocytes were sorted from the blood of MigR1-FlagYY1 reconstituted mice 14 weeks post reconstitution and crude cell lysates were made. Western blot was performed for detection of both endogenous and exogenous Flag tagged YY1. The upper band indicates Flag-tagged exogenous YY1 and the lower band indicates endogenous YY1. (TIF) [file pone.0030656.s001.tif]

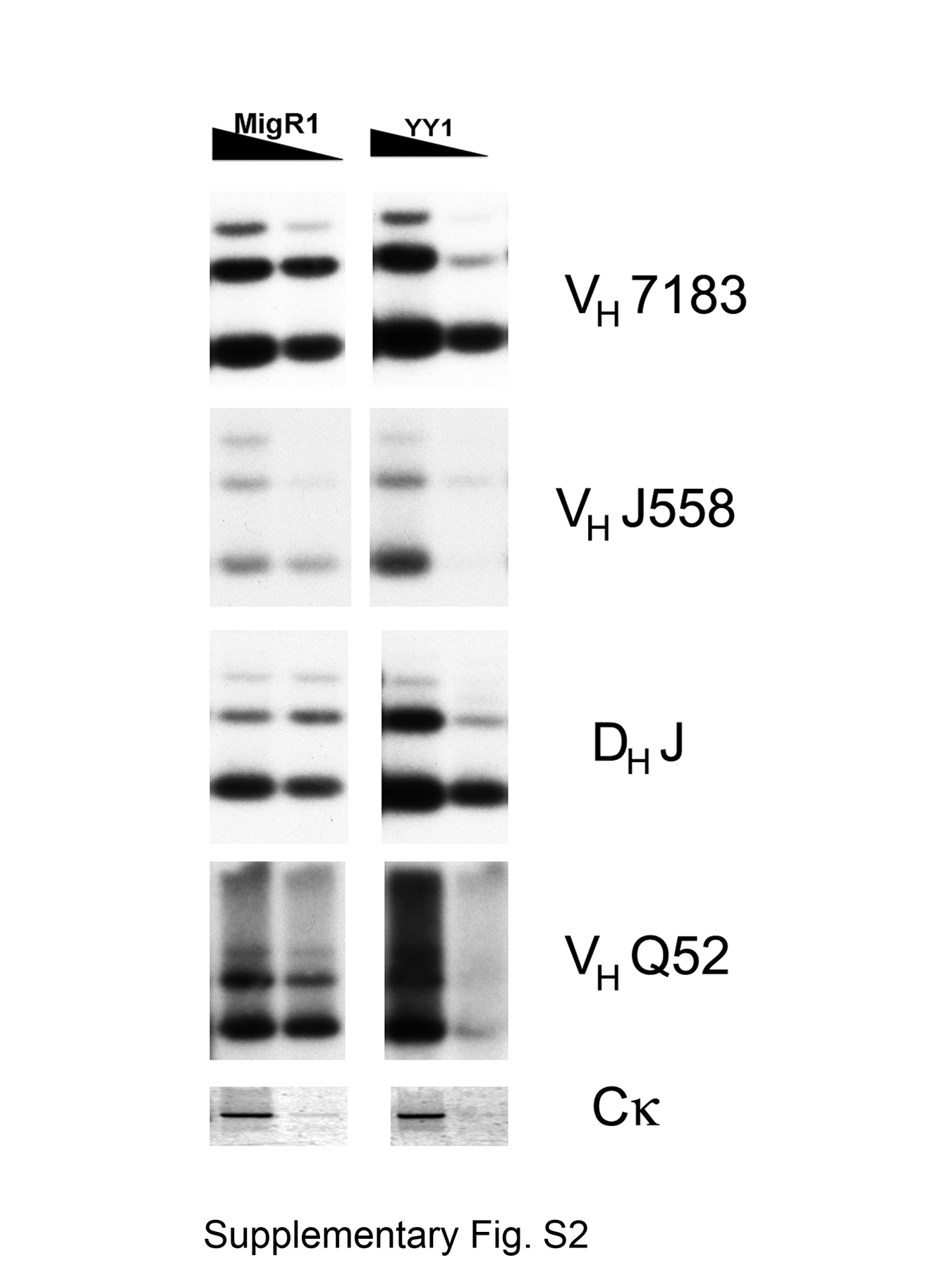

Supplement: Figure S2 — VDJ rearrangements are similar in MigR1 vector and MigR1-FlagYY1 transduced B cells from reconstituted animals. Rearrangement of various VH gene families is shown comparing mice reconstituted with MigR1 vector alone or MigR1-FlagYY1. V gene and DJ rearrangements are not altered by YY1 expression. (TIF) [file pone.0030656.s002.tif]

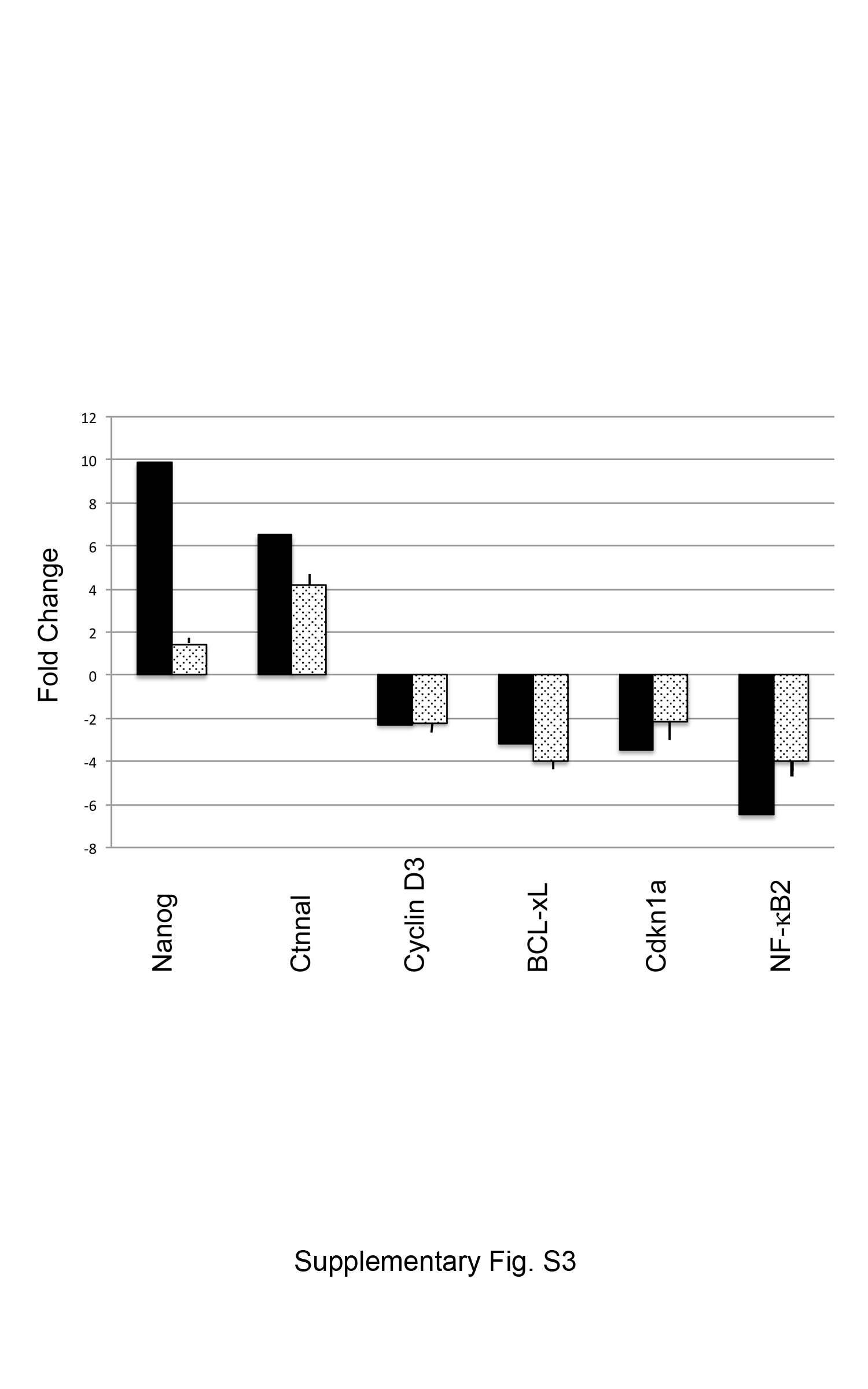

Supplement: Figure S3 — Confirmation of YY1 overexpression microarray results by RT-PCR. Microarray results (black bars) shown as fold change increase or decrease of MigR1-FlagYY1 transduced 38B9 cells relative to MigR1 vector alone, are compared with fold changes measured by RT-PCR (stippled bars). Error bars show the standard deviation of the mean. All transcripts matched closely by the two methods except for nanog expression which was induced to a much lower level as determined by RT-PCR. (TIF) [file pone.0030656.s003.tif]

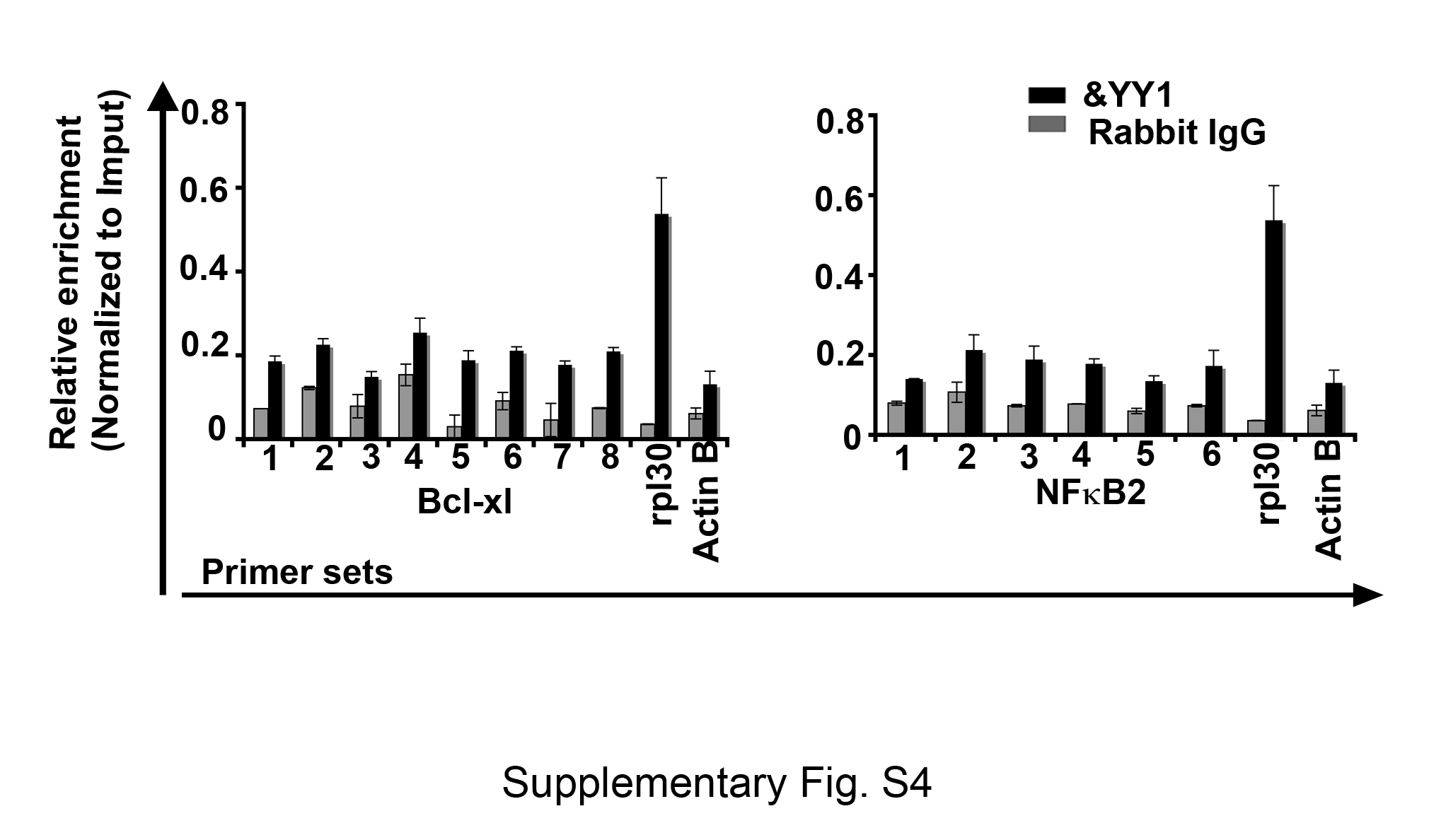

Supplement: Figure S4 — YY1 does not bind at the Bcl-xl or NFκB2 promoter areas. Chromatin made from murine 38B9 pro-B cells was immunoprecipitated with YY1 antibody or rabbit IgG control antibody. ChIP PCR was performed to detect the binding of YY1 at Bcl-xl or NFκB2 promoter areas. 8 sets of primers were designed to cover 1 kb of upstream promoter sequence of the Bcl-xl gene, and 6 sets of primers were designed to cover the NFκB2 promoter. RpL30 was used as a positive control for YY1 binding, and beta-actin was used as a negative control for YY1 binding. The mean and standard deviation are shown. (TIF) [file pone.0030656.s004.tif]
