# Supplementary material for: Increased Expression of PcG Protein YY1 Negatively Regulates B Cell Development while Allowing Accumulation of Myeloid Cells and LT-HSC Cells
Source: PLoS One. 2012 Jan 23;7(1):e30656. doi: 10.1371/journal.pone.0030656 (PMC3264595; doi:10.1371/journal.pone.0030656)
Supplement: Table S1 — Real-Time PCR Primers. (DOCX) [file pone.0030656.s005.docx]

**Supplementary Table S1**

**Real-Time PCR Primers**

**Ctnnal**

F primer GGGCTGCCTGGTCAGCTGTG

R primer GGCTGCACCTGTCCCTTCCC

**Bcl-xl**

F primer ACCACTACATGCAACTCACG

R primer ACCTCACTCAATGGCTCTTG.

**Cdkn1a**

F primer GCCGCGGTGTCAGAGTCTAGG

R primer GGGACCGAAGAGACAACGGCAC

**NF-κB2**

F primer ATCTGAGCATTGTACGGCTG

R primer TGTGAACTGTTTGGAGTCCG

**Cyclin D3**

| F primer | GGAAGCCTTTGCTTGCATGCACA |  |
| --- | --- | --- |
| R primer | AATGGCCGCTCGCTCCCCTA |  |

**Nanog**

| F primer | ACATGCTGGGCACCAACTCAACT |
| --- | --- |
| R primer | TGCACCTCACTGTCTCCAAAGCC |

**Gapdh**

F primer CTGAGGACCAGGTTGTCTCC

R primer GCCTCTCTTGCTCAGTGTCC
