# Supplementary material for: Increased Expression of PcG Protein YY1 Negatively Regulates B Cell Development while Allowing Accumulation of Myeloid Cells and LT-HSC Cells
Source: PLoS One. 2012 Jan 23;7(1):e30656. doi: 10.1371/journal.pone.0030656 (PMC3264595; doi:10.1371/journal.pone.0030656)
Supplement: Table S2 — Primers used for ChIP analyses. (DOC) [file pone.0030656.s006.doc]

Supplementary Table S2 Primers used for ChIP analyses.

| Gene | Forward primer | Reverse Primer |
| --- | --- | --- |
| Bcl-xl | CAGGCAACTAGGGTAAGGA | GAGTGGCACTATTTGGAGG |
|  | GTCTTTAATCCCAGCACTT | GACCAGACCACGAGTAAGC |
|  | AGGTCCCATGATCTAACAC | TCTCCTTAGCCTGACAACT |
|  | AGGGTCCATTCCGATCTAG | GTGTTATTAAACCAGCCAAA |
|  | TGTCCTCAGACCTCCATAT | TAGTCAGGCATTCTACCAA |
|  | TTTGCCGTCCCGCTTCCGTG | AGGCAACCGCCCTCCCTCAG |
|  | GAAGTCCCTTTAGGGTTTCG | CGGAGGTATGGGTTTAGTGTA |
|  | TTGTACCTGCTTGCTGTCG | ATTGTTTCATCCGCCTCTT |
|  | TTATGCCAGTCTGGTCTAT | CTTGAAGGCTTTATTGTGC |
|  | CCGCTTCTGCTTCTGACTT | CTTCTTCTTCTTCCCACCC |
| NFκB2 | AGCCACAGACGGGTTAGAG | ATGCCATCCATACACTTCCTT |
|  | GCCCAACCTGTAATCTATG | TTCCTTCTTATCTCCCACC |
|  | GAGCCTCCCACCCACTTTA | CACGCAAATCTGTCCAAAG |
|  | TTGTGAACCCGCCACTTAC | CATCTAGCGGCCTTTCTGG |
|  | GGAAAGAAGTCGGAACCAGA | CCAGCCTGTCATCTAAAGTCC |
|  | ACATCGTGAGTGAACAGAG | TGAAAGATTTTGCTGGGAT |
|  | CTATTCCCAAGAACCCACG | GCAACTGCGAATGCTGTCT |
| RpL30 | AGCAACCAACTACCGCAGACTACT | ATCCAGAGCGTCAAACACCAGCTA |
| Actin-B | aggcatggagtcctgtggtatc | AGCCACAGGTCCTAAGGCCAG |
